# Characterization of Gold-Sputtered Zinc Oxide Nanorods—a Potential Hybrid Material

**DOI:** 10.1186/s11671-016-1245-8

**Published:** 2016-01-19

**Authors:** Veeradasan Perumal, Uda Hashim, Subash C. B. Gopinath, Haarindraprasad Rajintra Prasad, Liu Wei-Wen, S. R. Balakrishnan, Thivina Vijayakumar, Ruslinda Abdul Rahim

**Affiliations:** Biomedical Nano Diagnostics Research Group, Institute of Nano Electronic Engineering (INEE), Universiti Malaysia Perlis, 01000 Kangar, Perlis Malaysia; School of Bioprocess Engineering, Universiti Malaysia Perlis, 02600 Arau, Perlis Malaysia

**Keywords:** Zinc oxide, Gold, Nanorods, Dopant, Impedance, Nanostructure

## Abstract

Generation of hybrid nanostructures has been attested as a promising approach to develop high-performance sensing substrates. Herein, hybrid zinc oxide (ZnO) nanorod dopants with different gold (Au) thicknesses were grown on silicon wafer and studied for their impact on physical, optical and electrical characteristics. Structural patterns displayed that ZnO crystal lattice is in preferred *c*-axis orientation and proved the higher purities. Observations under field emission scanning electron microscopy revealed the coverage of ZnO nanorods by Au-spots having diameters in the average ranges of 5–10 nm, as determined under transmission electron microscopy. Impedance spectroscopic analysis of Au-sputtered ZnO nanorods was carried out in the frequency range of 1 to 100 MHz with applied AC amplitude of 1 V RMS. The obtained results showed significant changes in the electrical properties (conductance and dielectric constant) with nanostructures. A clear demonstration with 30-nm thickness of Au-sputtering was apparent to be ideal for downstream applications, due to the lowest variation in resistance value of grain boundary, which has dynamic and superior characteristics.

## Background

Advances in nanotechnological approaches have provided an insight to nanocreations and manipulation of various nanomaterials to yield unique metal nanostructures having interesting properties and functions [1–3]. In the past, special attentions have been paid to make nanostructures for fine-tuning their properties towards the development of sensing substrates [4–6]. Among the different nanohybrid structures, involvements of metal oxides have elevated a step ahead in different applications [7–10]. Nanoparticle made from metal oxides proved for their participation in sensing applications, especially for bio-recognition [11, 12]. Oxide groups reside in the nanomaterials/nanoparticle prepared by metal oxide impart improvement in sensitivity of biosensors [13]. With the explored metal oxides, zinc oxide (ZnO)-based nanostructures have recently been aroused much interest due to its unique optical and electrical properties [14, 15].

ZnO has been widely used as material for semiconductor, because of its appealing characteristics, such as large exciton binding energy (60 meV). Special interests with ZnO usage have been taken to be used for electro-optical devices. Moreover, due to low cost, simplicity in fabrication and high electron mobility, optoelectronic devices rely on ZnO nanostructures (nanorods, nanowires and nanoflowers) [16–18]. Additionally, ZnO is stable at low and higher pH extremes and an ideal material for functionalization with biological and chemical compounds [11, 15]. With a property of excellent surface-to-volume ratio, ZnO nanohybrid has considered for improved catalytic activity. On the other hand, metal particles such as gold nanoparticle (AuNP) are shown to have high electron affinity; between AuNP and metal oxides, high Schottky barrier can be produced [19, 20]. Similar to ZnO, Au has been accepted as suitable material for biocompatibility, high conductivity and surface chemical functionalization [21–23]. Considering all these vital things, it is a wise approach to make ZnO and Au hybrid for creating nanostructures to be used for a wide range of applications.

In general, there are two major techniques that have been in nanofabrication of metal oxide nanostructures: “top-down” and “bottom-up”. Top-down approach is not a promising method because it contains some limitations such as low yield assembly, large-scale uniformity and repeatability issues, whereas bottom-up approach owns its superiority compared to top-down approach in terms of photolithography and is capable of producing various nanostructures with high yield, less defect and better range ordering [24]. ZnO nanostructures prepared by bottom-up approach are catalytically synthesized by chemical vapour deposition (CVD) and vapour liquid solid (VLS) methods, where structures are assembled from their atomic level [24, 25]. Hence, ZnO nanostructure from bottom-up fabrication approaches has been preferred as it possesses unique physical, optical and electrical properties, which are highly suitable for downstream applications.

In the present study, we demonstrated a simple and facile route to synthesis Au-sputtered ZnO nanorods, and the thickness of Au-sputtering is tuned to investigate physical, optical and electrical properties of ZnO nanorods on the interdigitated electrode (IDE). Currently, there are only limited numbers of research articles highlighting the impedance spectroscopic analyses on hybrid materials. Herein, impedance spectroscopy tool was employed to investigate on Au-sputtered ZnO nanohybrid. By the addition of AuNPs to ZnO nanostructures, it leads changes in conduction and polarization mechanism, which are clearly addressed.

## Methods

### Au-Interdigitated Electrode Fabrication

**Fig. 1:**
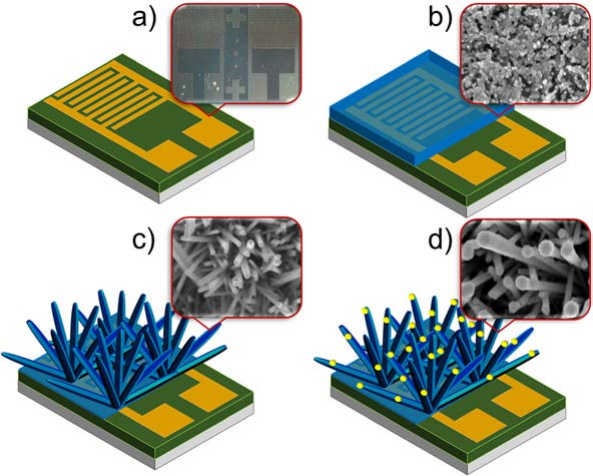
Schematic illustration shows the steps involved in the synthesis interdigitated electrode (IDE) coated with Au-sputtered ZnO-NRs. **a** Fabricated IDE. **b** ZnO thin film coating. **c** Hydrothermal growth of ZnO-NRs. **d** Sputtering different thicknesses of Au

ZnO nanorods were synthesized on a p-type silicon substrate. The silicon wafer (substrate) was cleaned using RCA1 and RCA2, which were prepared using hydrochloric acid (HCl; 37 %; J.T. Baker); aqueous ammonia (NH_4_OH; 30 %; J.T. Baker), hydrogen peroxide (H_2_O_2_; 30 %; J.T. Baker) and deionized water were used to remove organic and inorganic contaminations. Buffered oxide etchant (BOE; 6:1; J.T. Baker) was used to remove native oxide layer on the wafer surface [26, 27]. Using a lift-off process, IDE device with a 7 mm × 5 mm dimension was patterned using negative resists (NR7-6000PY; Futurrex.) on the SiO_2_/Si substrate [28]. A thermal evaporator (Auto 306 Thermal Evaporator; Edwards High Vacuum International, Wilmington, MA, USA) was used to deposit a titanium/Au (500/3000 Å) layer on the SiO_2_/Si substrate. Eventually, the negative photoresist sacrificial layer formed was removed using acetone. In this work, IDE with 16 fingers was fabricated, where the width and length of each finger was 0.1 and 3.9 mm, respectively, and the spacing between the two adjacent fingers was 0.1 mm. The fabricated IDE is shown in Fig. 1.

### ZnO Nanorods Synthesis

ZnO nanorods (ZnO-NRs) were prepared as described in our previous report [14]. Briefly, 8.78 g of Zn(CH_3_COO)_2_·2H_2_O (98 %; Sigma-Aldrich) was dissolved in 200 ml of ethanol solvent (EtOH; 99.99 %; J.T. Baker) (ZnO seed solution solgel). The concentration of ZnO was kept constant as 0.2 M. The mixed solution was then vigorously stirred with a magnetic stirrer at 60 °C for 30 min. The stabilizer, monoethanolamine (MEA; 99 %; Merck), was added drop by drop to the ZnO solution with constant stirring for 2 h. Finally, the transparent and homogenous solution was stored for aging at room temperature. The aged ZnO solgel was deposited on the IDE device by using a spin coating technique at a speed of 3000 rpm for 20 s. The deposition process of the seed layer was repeated for three times to get a thicker ZnO thin film. For each deposition process, the coated ZnO thin films were dried at 150 °C for 20 min to remove the organic residuals that might exist on the ZnO thin films. The coated ZnO thin films were then annealed in a furnace under ambient air at 300 °C for 2 h to get highly crystallized ZnO. For the hydrothermal growth of ZnO nanofilm, the prepared substrate with the coated seed layer was submerged backward inside the growth solution using a Teflon sample holder. Equal concentration (25 mM) growth solution was prepared by mixing both zinc nitrate hexahydrate (99 %; Sigma-Aldrich) and hexamethylenetetramine (99 %; Merck) in deionized water. The growth process was completed inside an oven at 93 °C for 5 h. The prepared hydrothermally grown ZnO nanofilm was cleaned with isopropanol and deionized water to remove residual salts prior to annealing in a furnace under ambient air at 300 °C for 2 h.

### Au-Decorated ZnO Nanorods Preparation

ZnO-NR-Au nanohybrids were prepared using a sputtering method. To form the ZnO-NR-Au nanohybrids, 10, 20, 30 and 40 nm Au wetting layers were physically deposited by a sputter coater (EMS550X) with Au target and a rotating stage. The detailed experimental conditions were as follows: electric current was maintained at 25 mA for 2–8 min with vacuum pressure of argon process level at 10^−2^ mbar. This process allowed us to obtain Au-decorated ZnO-NR forming ZnO-NR-Au nanohybrids. Figure 1a–d shows the schematic illustration of steps involved in the synthesis of Au IDE coated with Au-sputtered ZnO-NRs.

### ZnO-NR-Au Hybrids Material Characterization

The morphology and structural properties of ZnO-NR-Au nanohybrid samples were investigated under field emission scanning electron microscopy (FESEM; Carl Zeiss AG ULTRA55, Gemini). High-resolution transmission electron microscopy (HRTEM) image and selected area electron diffraction pattern (SAED) of ZnO-NR-Au nanohybrids were acquired using PHILIPS, CM-200 TWIN with an incident energy 200 keV. X-ray diffraction (XRD; Bruker D8, Bruker AXS, Inc., Madison, WI, USA) with a Cu Kα radiation (*λ* = 1.54 Ǻ) was used to study the crystallization and structural properties of the ZnO-NR-Au nanohybrids. The material composition was analysed using X-ray photoelectron spectroscopy (XPS) (Omicron Dar400, Omicron, Germany). The chamber pressure was maintained at 2.4e−10 Torr throughout the measurement. The obtained peak was deconvolution using CasaXPS software. In addition, the optical and luminescence properties of ZnO-NR-Au nanohybrids were studied through photoluminescence (PL; Horiba Fluorolog-3, HORIBA Jobin Yvon Inc., USA). The PL spectra of the sample were recorded at different angles and positions to assure the result is not influenced by sample non-homogeneity. The impedance spectroscopy measurements were taken with applied AC amplitude of 1 V RMS in the frequency range of 1 Hz to 100 MHz using Novocontrol Alpha high-frequency analyser (Hundsangen, Germany). All the measurements were performed at room temperature.

## Results and Discussion

### Morphological Features of Au-Sputtered ZnO Thin Film

#### FESEM Observations

**Fig. 2:**
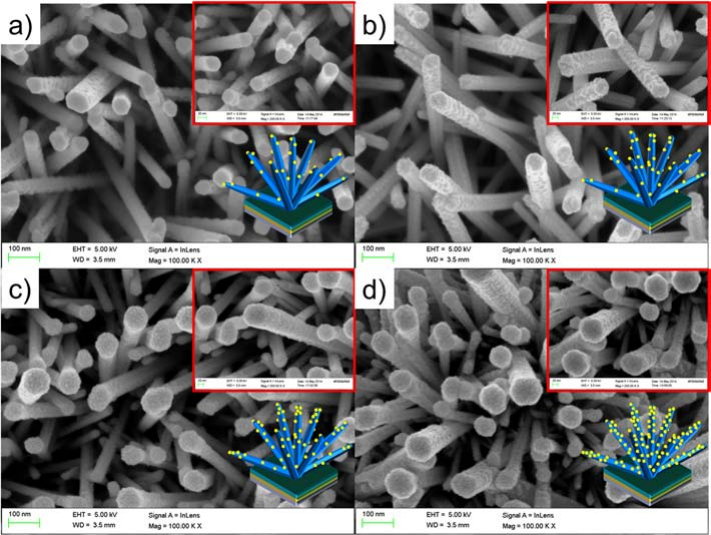
FESEM images of ZnO-NRs sputtered with different thicknesses of Au. **a** 10, **b** 20, **c** 30 and **d** 40 nm. The *inset* shows the magnified morphological observation of ZnO-NRs sputtered with different thicknesses of Au

The FESEM image depicting the surface morphology with various thicknesses of Au-sputtered ZnO nanorods is shown in Fig. 2a–d. The FESEM image displaying ZnO-NRs on the entire substrate surface was grown in high density. A careful observation on FESEM images in Fig. 2a–d revealed that the ZnO-NRs with hexagonal shape were elongated and protruding from the surface with average length of 1–2 μm and average diameter of 50–100 nm. As shown in Fig. 2a–d, it can be seen that the appearance of ZnO-NRs is surrounded by spherical nanoparticles of Au (bright spot). Clear agglomerations of AuNPs were observed in all surface of ZnO-NRs (Au-sputtered ZnO-NRs) as the sputtering thickness increased, demonstrating their homogeneity.

#### TEM Observations

**Fig. 3:**
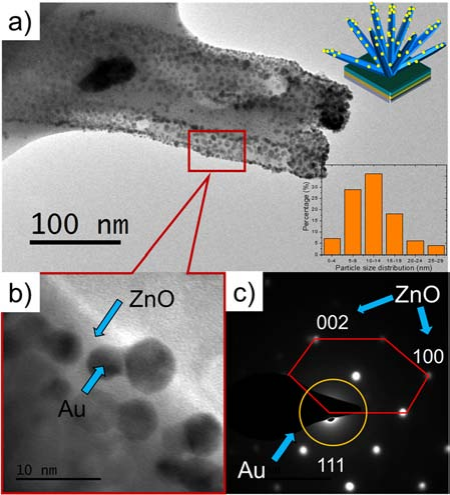
TEM images of ZnO-NRs sputtered with different thicknesses of Au. **a** Typical TEM micrograph of Au-sputtered ZnO-NRs and the *inset graph* is a histogram illustrating the particle size distributions. **b** High-resolution TEM image showing ZnO/Au nanohybrids. **c** Selected area electron diffraction pattern

TEM analysis was carried out to study further about the structural pattern of Au-sputtered ZnO-NRs. Figure 3 depicts low and high magnifications of TEM images and the selected area of electron diffraction pattern from fabricated Au-sputtered ZnO-NRs with a sputtered thickness of 30 nm. The low magnification of the TEM image on the small scraps of the specimens showed a clear visualization of the spherical, dumbbell and irregularly shaped structures of AuNP exhibiting a dark contrast, which are relatively well deposited on the surface of ZnO-NRs (Fig. 3a). The inset of Fig. 3a shows the size distribution of sputtered AuNPs with a range of 4–29 nm (minimum and maximum) with a sputtered thickness of 30 nm. Figure 3a also depicts the TEM image of a “blunt-ended” Au-sputtered ZnO-NRs, revealing the average diameter of 100 nm, which is in consistent with the observed FESEM results. High-resolution TEM image of the region is denoted in Fig. 3a, b, in which the spherical and dumbbell structures of AuNP are observed as dark spots and attached to the smooth ZnO-NRs with diameter of 5–10 nm, which is inconsistent with size distribution data. Figure 3c is showing SAED pattern of the Au-sputtered ZnO-NRs, with a hexagonal spot patterns corresponding to (100) and (002) planes and crystallography plane of AuNP indexed to (111), which indicates the presence of a highly crystalline ZnO wurtzite structure hybrid with sputtered AuNPs [29–31]. The SAED patterns suggested that ZnO-Au hybrid structure on Au-sputtered ZnO-NRs co-exist in the face-centred cubic structure.

### Structural Characterizations

#### XRD Analysis

**Fig. 4:**
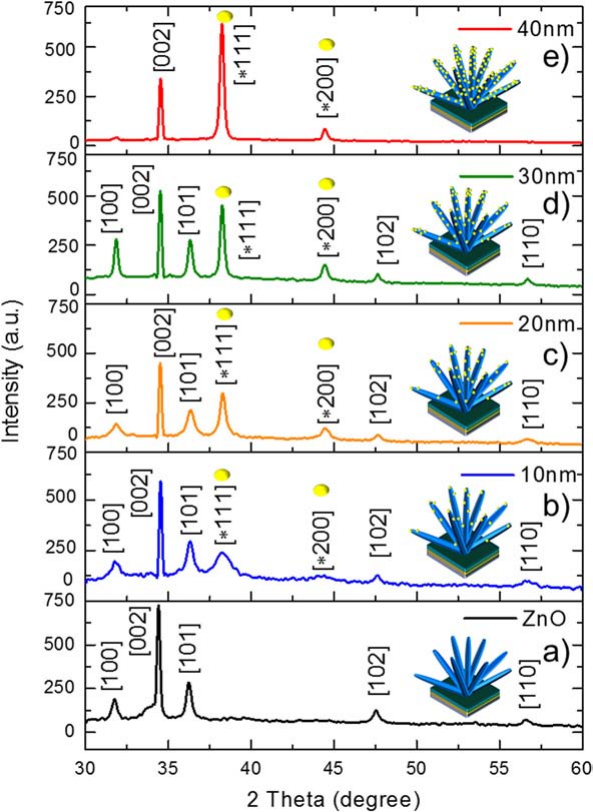
X-ray diffraction pattern of ZnO-NRs sputtered with different thicknesses of Au. **a** Bare ZnO-NRs; **b** 10-, **c** 20-, **d** 30- and **e** 40-nm Au-sputtered ZnO-NRs

X-ray diffraction analysis was carried out to examine the crystal quality, size, orientation and morphology of fabricated Au-sputtered ZnO-NRs. Figure 4a shows a typical X-ray diffraction (XRD) pattern of the as-synthesized ZnO-NRs coated on the silicon substrate. Figure 4b–e shows the XRD pattern of ZnO-NRs with various Au thicknesses (10–40 nm). As shown in Fig. 4, the XRD spectra of Au-sputtered ZnO-NRs were compared to that of pure ZnO-NRs. The diffraction peaks are corresponding to Au and ZnO and were in agreement with those reported in the Joint Committee on Powder Diffraction Standards (JCPDS) reference spectra (no. 36-1451) for standard ZnO and (no. 65-2879) bulk Au, which indicates that the obtained spectra are in good agreement with the standard JCPDS data cards. The obtained XRD patterns exhibited reflection peaks at 31.86° (100), 34.49° (002), 36.34° (101), 47.63° (102) and 56.65° (110). Thus, the resultant planes in Fig. 4a can be indexed to the hexagonal wurtzite phase of a ZnO nanostructure [32]. Interestingly, two additional diffraction peaks were observed in Fig. 4b–e compared to the spectra of the pure wurtzite ZnO-NRs. The diffraction peaks located at 38.27° and 44.49° were assigned to (111) and (200) diffraction lines, respectively, which were indexed to the face-centred cubic Au structure [14, 33]. This result is in good agreement with the results observed by SAED. Here, (002) reflection appeared to be dominant for all the samples, which suggests that the ZnO-NRs preferred anisotropic growth along the [001] direction of the substrate [34, 35]. The observed diffraction peak corresponding to Au (111) and (200) exhibits gradual broadening with sharpening of the increased peak intensity as Au thicknesses were increased (10 to 40 nm). Au peak (111), which is shown in Fig. 4b–e with increased intensity, indicates significant growth of the Au grain sizes, due to the increment in Au thickness and Ostwald ripening. Ostwald ripening also contributes to the high interatomic forces that occur at larger Au thicknesses [36, 37]. The sharp and narrow shape of the peaks indicates that Au-sputtered ZnO nanocomposites have good crystalline quality. The crystallite structure of ZnO and AuNPs were estimated using the Scherrer equation. The crystallite sizes of the Au-sputtered ZnO-NRs, estimated from the broadening of XRD peaks, are about 57.14 nm, whereas AuNP has 7.6 nm. The result was in good agreement with the HRTEM result. The lattice parameters are indicating that the product was a composite material and no other impurities were found. Thus, resultant planes demonstrated that the pure hexagonal wurtzite ZnO structure were synthesized with high-quality crystals and *c*-axis alignment.

#### XPS Analysis

**Fig. 5:**
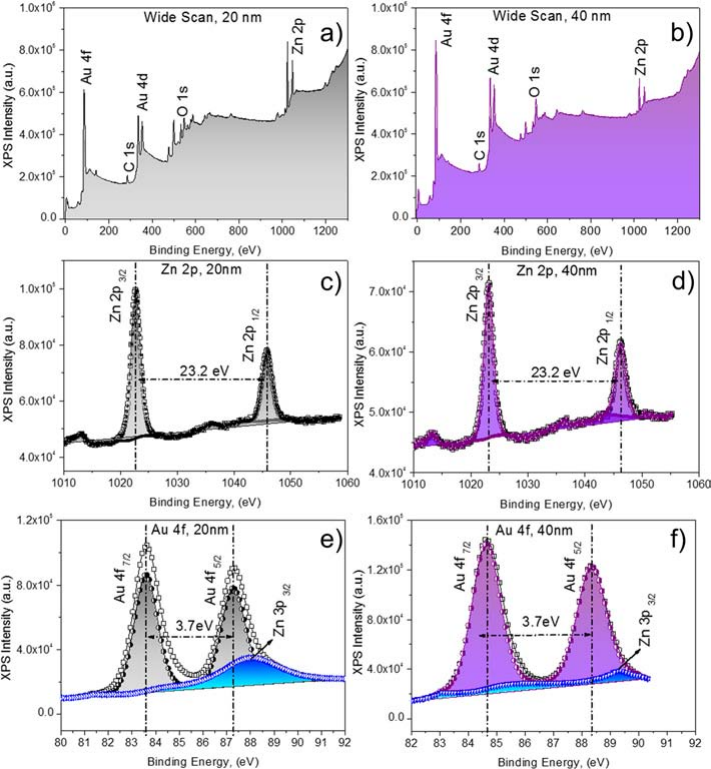
X-ray photoelectron spectroscopy data showing binding energies of 20- and 40-nm Au-sputtered ZnO-NRs. **a** Survey scan of 20 nm. **b** Survey scan of 40 nm. **c** Zn 2p, 20 nm. **d** Zn 2p, 40 nm. **e** Au 4f, 20 nm. **f** Au 4f, 40 nm

XPS studies were performed to investigate the elemental composition and its chemical state present in the outmost layer of the Au-sputtered ZnO-NRs. The wide XPS survey scan of the 20- and 40-nm Au-sputtered ZnO-NRs was shown in Fig. 5a, b. The wide scan contains the same photoelectron peaks that correspond to carbon (C), oxygen (O), zinc (Zn) and gold (Au) in the 20- and 40-nm Au-sputtered ZnO-NRs without any impurities (Fig. 5a, b). The binding energies were calibrated within an accuracy of 0.1 eV using C1s (284.6 eV). The corresponding Zn, Zn 2p and Au (Au4d and Au4f) XPS spectra that were acquired from the Au-sputtered ZnO-NRs and the wide scan are shown in Fig. 5c–f. As shown in Fig. 5c, d, Zn 2p XPS spectra of the 20- and 40-nm Au-sputtered ZnO-NRs contain two peaks corresponding to Zn 2p_3/2_ and Zn 2p_1/2_, which correlate the binding energies of 1022.63 and 1045.83 eV, respectively, due to the Zn^2+^ valance state [38–40]. The observed Zn 2p binding energy for Au-sputtered ZnO thin film exhibits a slightly positive shift of ∆*E*_zn_ = 1.13 eV, compared to the corresponding value for elemental Zn (1021.5 eV) [41, 42]. This result indicated that Zn and Au interacted to form a compound, due to the structural defects and oxygen vacancies in ZnO. Furthermore, the observed binding energy for Zn 2p revealed the nature of the oxidation state of Zn and no metallic Zn. Figure 5e, f shows the core level XPS spectra of Au 4f and Zn 3p for the 20- and 40-nm Au-sputtered ZnO-NRs. The binding energies for the 20-nm Au-sputtered ZnO-NRs at 83.75, 87.45 and 88.2 eV corresponding to Au 4f_7/2_, 4f_5/2_ and Zn 3p_3/2_, respectively, are shown in Fig. 5e. However, the photoelectron peak for the 40-nm Au-sputtered ZnO NRs corresponding to Au 4f_7/2_, 4f_5/2_ and Zn 3p_3/2_ has binding energies of 84.66, 88.34 and 89.26, respectively, as shown in Fig. 5f. The variation in Au 4f_7/2_ binding energy compared to that of bulk Au (84.0 eV) confirmed the strong electronic interaction between the AuNPs and ZnO [43, 44]. However, the intensity of Au photoelectron peaks (Au 4f_7/2_ and 4f_5/2_) increases as the sputtering thickness of Au increased from 20 to 40 nm, whereas the intensity of Zn peaks (Zn 2p_3/2_, Zn 2p_1/2_ and Zn 3p_3/2_) decreases, mainly due to the higher inelastic mean free path (IMFP) attributed to the layer of Au thickness adhering on the sample surface [45, 46].

### Optical Characterizations

#### PL Measurements

**Fig. 6:**
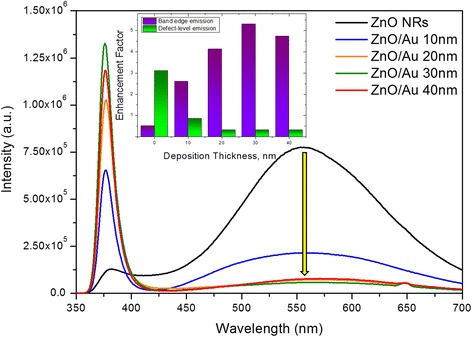
Photoluminescence spectra of ZnO-NRs sputtered with different thicknesses of Au with figure *insets* showing the corresponding variations of enhancement factors of UV emission with different deposition thicknesses of AuNPs

Figure 6 presents the PL characteristics manifested by ZnO nanorods and Au-sputtered ZnO nanorods. For ZnO nanorods, a weak near-band-edge UV emission at 375 nm and a broad, strong green emission peak at 560 nm were observed. In ZnO, the broad hump green emission corresponds to the structural defect, such as Zn vacancy (*V*_zn_) and/or oxygen vacancy (*V*_o_) [47, 48]. When bare ZnO sample were excited using 325 nm line of continuous-wave by He-Cd laser, only a fewer electron reached conduction band while the majority of the electron are trapped in the defect level. Thus, the electron in the defect level is readily recombined with the holes in the valence band of ZnO to give a broad hump over the visible emission [49, 50]. Moreover, the intensity of UV band emission was significantly reduced owing to lower recombination of electron-hole pairs from the conduction band. However, for the 10- to 40-nm Au-sputtered ZnO-NRs samples, the band edge emission intensity has been drastically increased. On the contrary, the green emission was significantly suppressed to the noise level as the sputtering thickness increases from 10 to 40 nm. Generally, the complete suppression of green emission and increased band edge emission are related to noble metal (Au), an introduction to semiconducting ZnO structure [51, 52]. The energy level of defect states and Fermi level of Au are very close to each other. Therefore, the electron from the defect level of ZnO can transfer to the surface plasmons of Au, which significantly increase the electron density. Consequently, surface plasmons of Au were excited, prompting the electron to elevate to higher energy state than the conduction band of ZnO. These electrons could then easily transfer back to the conduction band of ZnO, which facilitates the recombination of electron-hole pairs in ZnO valance band [53–55]. As shown in the inset (Fig. 6), the enhancement factor of UV emission reaches a maximum value with 30 nm sputter thickness and then decreases with a further increase to AuNP thickness of 40 nm. Hence, with the sputtering thickness was increasing from 10 to 40 nm, Au particles grow larger and form a continuous film throughout the ZnO-NRs structures. In this case, the adsorption process will rule over the scattering so that radiation as photons into free space will be suppressed due to non-radioactive dispersion of the surface plasmon [56]. This phenomenon accounts for the attenuation of light emission (UV emission) for ZnO-Au after sputtering 40-nm AuNP. To conclude, the mechanism explains that the enhancement in band edge emission and suppression of defect emission could be achievable through AuNP sputtering to ZnO-NRs.

### Electrical Characterizations

#### Impedance Analysis

**Fig. 7:**
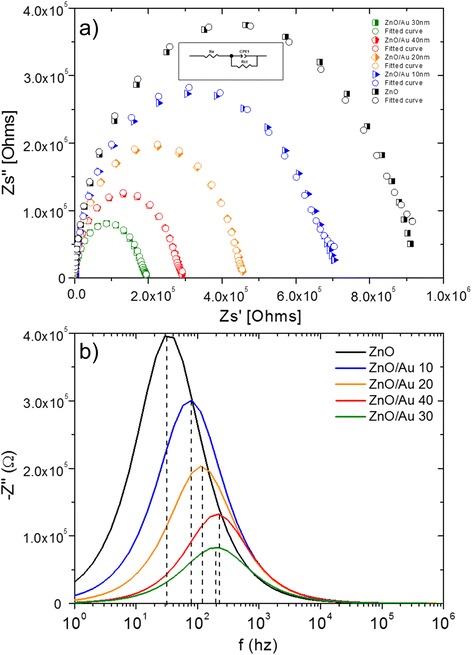
Impedance characterizations of ZnO-NRs sputtered with different thicknesses of Au. **a** Nyquist plot and **b** imaginary part showing the overall impedance

To investigate the effect on the conduction mechanism of sputtered AuNP layers of various thicknesses, AC impedance spectroscopic analyses were performed. Figure 7a depicts Nyquist plot consists of real (Z′) and imaginary (Z′′) in the complex impedance spectra of Au-sputtered ZnO-NRs. These plots represent the relaxation times and resistance related to bulk grain, grain boundaries and electrode interfaces in complex impedance plane. ZView software from Scribner Associates Inc. was used to model the impedance semicircle. Table 1 displays the simulation results with non-linear curve fitting for the measured impedance with an equivalent circuit. The obtained Nyquist plot can be expressed by Randles equivalent circuit (inset Fig. 7a), where the parameters Ra, Rct and CPE represent the resistance of bulk solution, charge transfer resistance and constant phase element, respectively. The semicircle in impedance spectra represents the interfacial charge transfer resistance, Rct, corresponding to the carrier transfer from IDE electrode. As shown in Fig. 7a, the semicircle (bare ZnO-NRs) exhibits very high Rct value (~0.916 MΩ). The result suggests that interfacial layer of bare ZnO-NRs has a lower electron transfer property owing to its low surface area. These variations were attributed to grain sizes and dipole dynamics [57, 58]. During the annealing treatment, oxygen molecules from the ambient atmosphere can be easily adsorbed on ZnO thin films owing to the large surface area-to-volume ratio of the ZnO thin film. Thus, adsorbed oxygen molecules trapped many electrons from the conduction band of ZnO at grain boundary surfaces, which leads to increase in resistance. Hence, sufficient numbers of trapped electrons give rise to a strong electric field, which affects the dipoles around the trap site, forming spatial charges [59, 60]. Therefore, the observed impedance for bare ZnO nanorods is the highest. There is a decrement in Rct values for 10-, 20- and 30-nm-sized Au-sputtered thicknesses (~0.703, 0.431, 0.101 MΩ) which were observed with Au-sputtered ZnO-NRs against the bare ZnO-NRs. The observed diameter decrement can be explained as a system in which the Fermi energy level of ZnO is lower than Au. This energy-level disparity is a consequence of the fact that the work function of ZnO is higher (5.2–5.3 eV) than Au (5.1 eV), resulting in an electron transfer from Au to ZnO until two systems reaches a dynamic equilibrium [61, 62]. However, a contrasting trend was observed with the 40-nm-thick sputtered AuNP layer (0.289 MΩ). When the sputtering thickness increased to 40 nm, Au particles were grown larger and form a continuous film where the ohmic characteristic of the film changes to rectifying characteristics. This phenomenon accounts for attenuation impedance variation for Au-sputtered ZnO after 40 nm sputtering AuNP. Figure 7b shows the imaginary part of impedance by plotting the imaginary (−Z′′) against the logarithm of the frequencies which exhibits the Debye-type peaks. It was obvious that the imaginary part of overall impedance decreases as ZnO-NRs sputtered with AuNP and the peak frequency shifted towards the higher frequencies when the thicknesses of Au were increased. Decrease in impedance imaginary part indicates that the conductivity increases, and the shifting indicates that Au thickness increases along the increment in relaxation time. Thus, increasing Au thickness results in overall decrease in impedance imaginary part which reflects the ease flow of the charge carriers to the AC electric field [57, 59].

**Table 1:** Parameters for ZnO nanorods sputtered with different Au thicknesses

Samples	Rct (MΩ)	CPE (nF)	*n* value
ZnO	0.92	13.58	0.89
ZnO/Au 10 nm	0.70	9.85	0.89
ZnO/Au 20 nm	0.43	7.27	0.90
ZnO/Au 30 nm	0.10	3.51	0.91
ZnO/Au 40 nm	0.29	4.38	0.91

## Conclusions

ZnO-Au hybrid nanorods were grown successfully using hydrothermal method sputtered with different thicknesses of Au and have superior structural, optical and electrical characteristics compared to bare ZnO-NRs. Complete characterization of this structure was clearly demonstrated for their ultimate high-performance sensing. To investigate the effect of sputtered AuNP layers on the conduction mechanism, AC impedance spectroscopic analyses were performed. The results showed that the impedance and dielectric constant were decreased with the thickness of AuNP seeding increased. These variations were attributed to the grain sizes and dipole dynamics [57]. A clear demonstration was shown with 30 nm thickness of Au, has a lowest variation in resistance value of grain boundary compared to other sizes fabricated, to be an optimal material for sensor. This study has shown an optimized ZnO/Au nanohybrid with complete characterizations, a tailored nanomaterial for downstream applications.
